# Molecular pathogenesis in granulosa cell tumor is not only due to somatic *FOXL2* mutation

**DOI:** 10.1186/s13048-014-0088-0

**Published:** 2014-09-06

**Authors:** Wen-Chung Wang, Yen-Chein Lai

**Affiliations:** Department of Obstetrics and Gynecology, Jen-Ai Hospital, Taichung, Taiwan; School of Medical Laboratory and Biotechnology, Chung Shan Medical University, No.110, Sec. 1, Chien Kuo N. Road, Taichung, 402 Taiwan

**Keywords:** Granulosa cell tumor, *FOXL2* mutation, Loss of heterozygosity, Array comparative genomic hybridization

## Abstract

Granulosa cell tumors are rare ovarian malignancies. Their characteristics include unpredictable late recurrent and malignant behavior. Recent molecular studies have characterized the *FOXL2* 402C > G mutation in adult-type granulosa cell tumor. In this study, we report an 80-year-old woman with a granulosa cell tumor arising from ovary. She presented with a huge pelvic mass with postmenopausal bleeding. No obvious intraperitoneal tumor implants were observed during operation. Final diagnosis was granulosa-theca cell tumor without capsule invasion. No recurrent disease was noted during 3-year post-operation follow-up period. Molecular studies showed a heterozygous *FOXL2* 402C > G mutation in the tumor by direct gene sequencing. In addition, DNA replication error, on analysis of the lengths of CAG repeats in androgen receptor gene, revealed defective DNA mismatch repair system in the granulosa cell tumor. We propose that the 402C > G mutation in FOXL2 is critical to the development of adult granulosa cell tumor. However, the malignant behavior of this tumor is driven by DNA mismatch repair deficiency. Unequal DNA copy numbers were noted on array comparative genomic hybridization. This implies that there is malignant potential even in the early stage of the granulosa cell tumor. Late malignant recurrence may be a late event of DNA repair function disability, not directly related to pathognomonic *FOXL2* mutation.

## Background

Sex cord-stromal tumors represent approximately 8% of all ovarian tumors [1]. The incidence is about 0.2 per 100000 women. The most frequently diagnosed tumor type within sex cord-stromal category is the granulosa cell tumor [1]. Granulosa cell tumors may be composed almost exclusively of granulosa cells, but more commonly also contain theca cells, lutein cells, and/or fibroblasts [2]. The granulosa cells in tumor may be arranged in trabecular, insular, grand like, microfollicular, macrofollicular or diffuse cellular patterns [3]. Granulosa cell tumors can be further categorized into two distinct subtypes, juvenile and adult forms [1]. Juvenile granulosa cell tumor is characterized by a modular or diffuse pattern, with follicle-like spaces of variable size and shape and striking cytologic atypia [3]. In some cases, these tumors cannot be distinguished from each other or from other malignant tumors. Recent studies of *FOXL2* (forkhead box L2) gene 402C > G (C134W) mutation may resolve the problems of adult-type granulosa cell tumor diagnosis [4]. Adult-type granulosa cell tumors are much more common and mostly occur in perimenopausal or early postmenopausal females [5].

Granulosa cell tumors are generally considered to have a better prognosis than epithelial ovarian tumors [1]. However, there is high rate of tumor recurrence late in life and approximately 80% of patients with advanced stage or recurrent tumors succumb to their disease [6]. Granulosa cell tumors with malignant potential comprise 1.0 percent of all ovarian cancers [1]. The characteristics associated with granulosa cell tumors cannot be explained by ploidy or p53 overexpression [7,8].

## Case report

**Figure 1:**
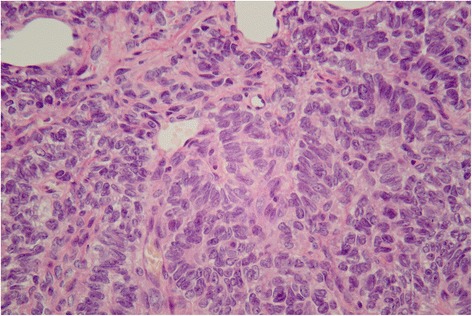
**Granulosa cell tumor: Histological cross-section after hematoxylin and eosin staining shows the adult granulosa cell tumor component.**

An 80-year-old woman presented with a clinically detectable pelvic mass. She subsequently underwent laparotomy for total hysterectomy and bilateral salpingo-oophorectomy. Gross examination demonstrated an ovarian mass with attached fallopian tube, measuring 11.0 × 8.0 × 7.0 cm, which was brownish in color and elastic. On cut, the ovary was yellowish in color and soft. Microscopically, the ovary revealed a granulosa-theca cell tumor characterized by granulosa cells in trabecular or cylindrical pattern and theca cells in spindle pattern (Figure 1). FIGO stage IA was supported by pathological report. Her blood inhibin A and B levels were 47.208 pg/ml and 92.473 pg/ml, respectively, immediately after operation and 15.533 pg/ml and 22.331 pg/ml, respectively, one month post-operation. No recurrent disease was noted during 3-year post-operation follow-up period. The Institutional Review Board of Chung Shan Medical University Hospital approved all procedures and informed consent was obtained prior to collecting her genetic material for the study.

**Figure 2:**
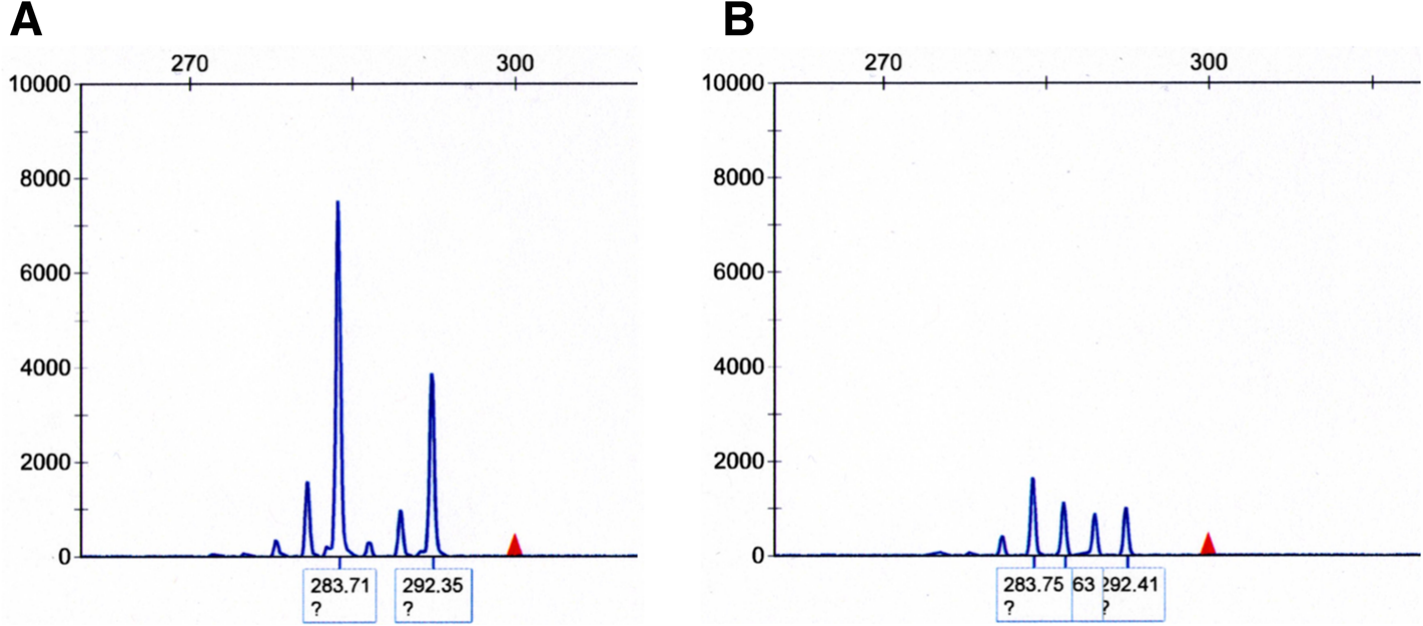
**Replication error was detected on analysis of the CAG repeats in androgen receptor gene by capillary gel electrophoresis.** DNA from tumor **(B)** is compared with DNA from normal cells **(A)** from the same patient.

**Figure 3:**
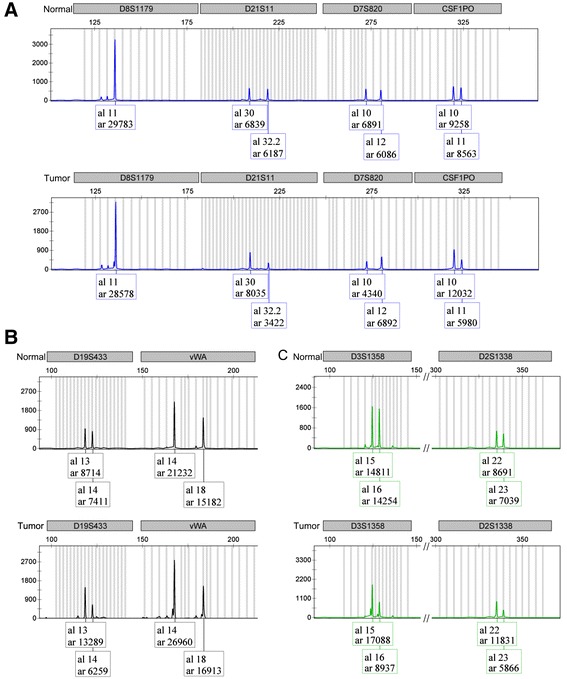
**LOH for a number of markers: Selective electrophoregram of the DNA profiles of blood and tumor tissue using short tandem repeats (STR) analysis with AmpFlSTR SGM Plus PCR amplification kit.** Colored panels are representative of the following: **(A)** STR loci of D8S1179, D21S11, D7S820, and CSF1PO; **(B)** STR loci of D19S433 and vWA; **(C)** STR loci of D3S1358 and D2S1338.

**Figure 4:**
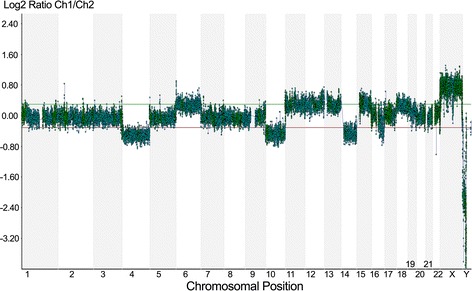
**Array comparative genomic hybridization (aCGH) analysis: Whole genome view on comparative genomic hybridization array (CytoChip Oligo Array) shows pathological genetic imbalances in many chromosomes in the tumor.**

We tested for the presence of *FOXL2* 402C > G mutation using PCR and direct sequencing according to the methods described by Schrader et al [9]. The nucleotide sequence analysis of *FOXL2* from the granulosa cell tumor section revealed heterozygous 402C > G mutation. In contrast, *FOXL2* sequences of genomic DNA isolated from the blood and normal tissue of this patient did not demonstrate this mutation. When compared with DNA from normal cells (Figure 2A), DNA replication error caused by slippage between the replication apparatus and the DNA template was detected in the granulosa cell tumor (Figure 2B), on analysis of the lengths of CAG repeats in androgen receptor gene, using published primers and experimental conditions [10]. In addition, we compared the DNA profiles of normal tissue and tumor tissue using short tandem repeats (STR) analysis according to the methods described by Wang et al [11]. The tumor showed loss of heterozygosity (LOH) for a number of markers (Figure 3 and Table 1). Moreover, based on array comparative genomic hybridization (CGH), pathological genetic imbalances were detected in the tumor on CytoChip Oligo Array (BlueGenome): monosomy 4 and 10; partial monosomy 14 with segmental deletions; and mosaic trisomy 6, 11, 12, 13, 15, and 18 with segmental duplications (Figure 4 and Table 2).

**Table 1:** **Loss of heterozygosity in 15 STR loci of the granulosa cell tumor**

**STR Loci**	**Location**	**Alleles**	**R** ^**a**^
TPOX	2p23-2per	8, 8	ND
D2S1338	2q35-37.1	22, 23	1.63^b^
D3S1358	3p21.31	15, 16	1.84^b^
FGA	4q28	22, 23	1.01
D5S818	5q21-31	11, 11	ND
CSF1PO	5q33.3-34	10, 11	1.86^b^
D7S820	7q11.21-22	10, 12	0.56^b^
D8S1179	8q24.1-24.2	11, 11	ND
TH01	11p15.5	9, 9	ND
vWA	12p12-pter	14, 18	1.14
D13S317	13q22-31	10, 13	0.94
D16S539	16q24-qter	9, 11	1.11
D18S51	18q21.3	14, 18	1.06
D19S433	19q12-13.1	13, 14	1.81^b^
D21S11	21q11.2-q21	30, 31.2	2.12^b^

^a^R = (area T1/area T2)/(area N1/area N2); ^b^Loss of heterozygosity (LOH) is positive when R ≥ 1.25 or ≤ 0.8 (ie., 20% change).

**Table 2:** **The deleted or duplicated clones and their physical location in the granulosa cell tumor**

	**ISCN nomenclature**	**Type**	**Size (bp)**
1	arr 4p16.3q35.2(37,152-190,896,645)×1	LOSS	190,859,493
2	arr 6p25.3q27(163,113-170,921,060)×2 ~ 3	GAIN	170,757,947
3	arr 10p15.3q26.3(136,391-135,434,149)×1	LOSS	135,297,758
4	arr 11p15.5q25(196,990-134,868,378)×2 ~ 3	GAIN	134,671,388
5	arr 12p13.33q24.33(230,451-133,773,499)×2 ~ 3	GAIN	133,543,048
6	arr 13q12.11q34(20,407,324-115,092,619)×2 ~ 3	GAIN	94,685,295
7	arr 14q11.2q32.33(20,608,246-107,287,476)×1	LOSS	86,679,230
8	arr 15q11.1q26.3(20,686,219-102,383,444)×2 ~ 3	GAIN	81,697,225
9	arr 16q11.2q24.3(46,500,771-90,148,364)×1 ~ 2	LOSS	43,647,593
10	arr 18p11.32q23(148,993-78,012,800)×2 ~ 3	GAIN	77,863,807

## Discussion

Recent studies have shown that *FOXL2* 402C > G is a diagnostic characteristic of adult-type granulosa cell tumor [4]. However, little is known about how a granulosa cell tumor undergoes indolent course with late recurrent behavior [12]. We report a case of adult-type granulosa cell tumor with *FOXL2* 402C > G mutation in an 80-year-old woman, in which one allele was normal and one was mutant. This mutation was not observed in the blood and normal tissue samples of this patient. Histological diagnosis is compatible with adult-type granulosa cell tumor in this case. RNA transcription arrays from granulosa cell tumor cell line have indicated that alteration involves cell death, proliferation and tumorigenesis [13]. However, the mechanism is not clear.

DNA replication error on analysis of the lengths of CAG repeats in androgen receptor gene (Figure 2B) is consistent with a previous study suggesting that a DNA mismatch repair deficiency contributes to the pathogenesis of granulosa cell tumors, and that this deficiency is an early event in their development and/or progression [14]. LOH for a number of markers (Table 1 and Figure 3) and pathological genetic imbalances throughout the genome (Table 2 and Figure 4) imply that a defective upstream regulatory gene is involved in this condition. The *MLH1* gene that encodes a component of the mismatch repair system located near the D3S1358 locus showed LOH (Table 1). This implies that the upstream regulatory mechanisms leading to increased DNA replication errors, LOH, and genetic imbalances are associated with defect(s) in global gene regulation.

Our assumption is that a genetic alteration in mismatch repair system occurs before *FOXL2* 402C > G mutation. After this mutation is initiated, granulosa cell tumor develops in early tumorigenesis. The genomic imbalances on array CGH in this study were inconsistent with the results of other array and CGH studies [15]. However, there was a similar trend of genetic imbalances with a loss of 16q. These genetic imbalances may contribute to late tumorigenesis. Our hypothesis is that a DNA repair system failure induces *FOXL2* 402C > G mutation, followed by granulosa cell tumor development. The same mechanism randomly causes further mutations of tumor suppressor genes or oncogenes, resulting in late recurrence and unpredictable malignant behavior of granulosa cell tumor. However, we cannot dismiss the possibility that the amino acid changing mutation in *FOXL2* 402C > G is the driver mutation that leads to subsequent genomic alterations in granulosa cell tumor pathogenesis.

## Conclusions

Adult-type granulosa cell tumors are associated with *FOXL2* 402C > G mutation. In addition to this unique *FOXL2* mutation, we found DNA replication error and loss of heterozygosity in this case. DNA mismatch repair system failure appears likely in this patient. In such a case, early detection allows for treatment of benign tumor. Although this study could not elucidate the exact mechanism for the development of granulosa cell tumor, it does suggest the need to incorporate DNA mismatch repair system examination into the clinical management of patients with granulosa cell tumor.

## Consent

Written informed consent was obtained from the patient for publication of this Case report and any accompanying images. A copy of the written consent is available for review by the Editor-in-Chief of this journal.

## Abbreviations

aCGH: Array comparative genomic hybridization
FIGO: The International Federation of Gynecology and Obstetrics
*FOXL2*: Forkhead box L2
LOH: Loss of heterozygosity
*MLH1*: MutL homolog 1
PCR: Polymerase chain reaction
STR: Short tandem repeat
